# Supplementary figures and images for: Limited reciprocal surrogacy of bird and habitat diversity and inconsistencies in their representation in Romanian protected areas
Source: PLoS One. 2022 Feb 11;17(2):e0251950. doi: 10.1371/journal.pone.0251950 (PMC8836316; doi:10.1371/journal.pone.0251950)

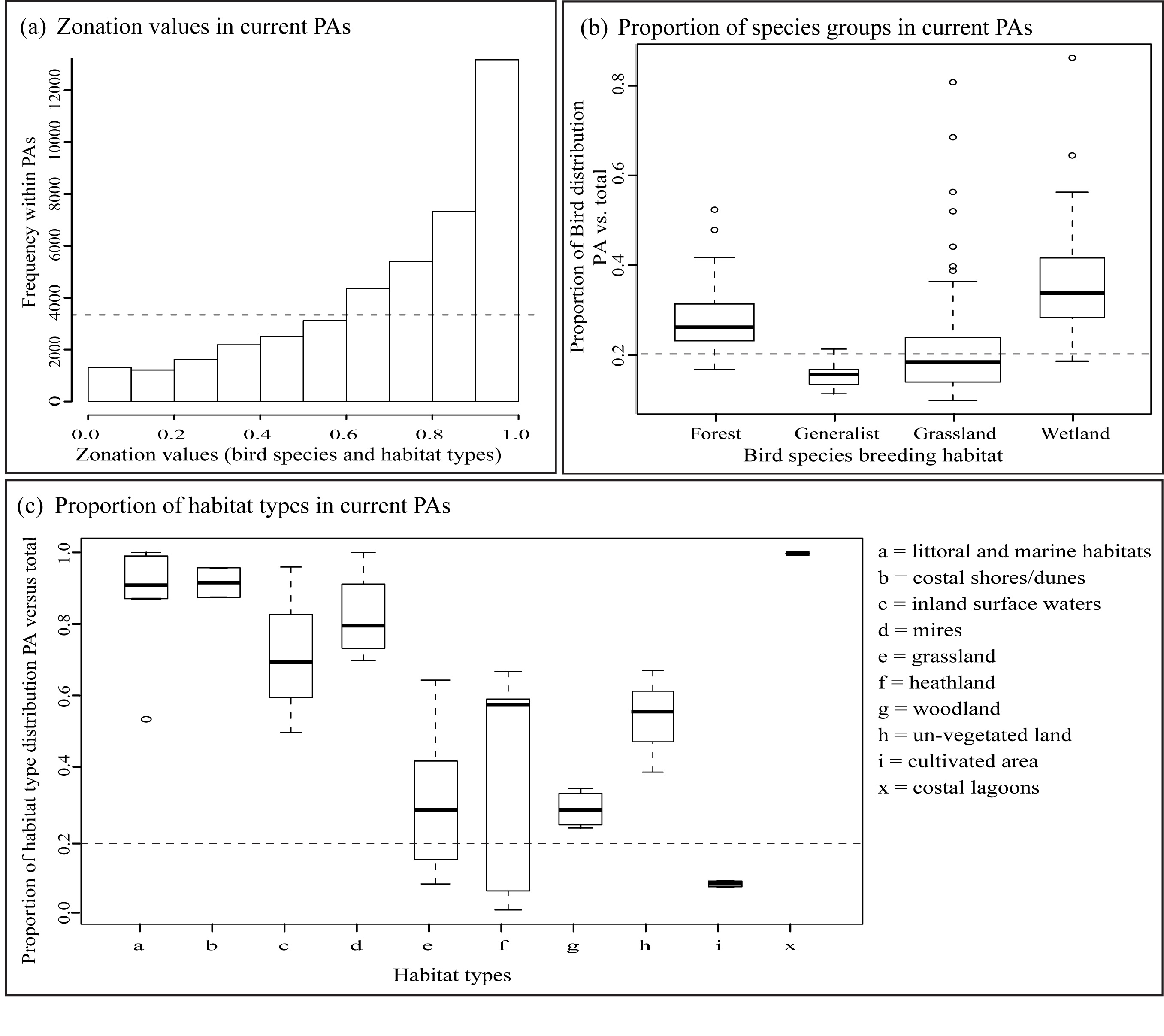

Supplement: S1 Fig — (a) Barplot of conservation values of areas in current reserves. The horizontal dashed line indicates the expected frequency of each conservation value (freq = 3338.6), had the current PAs be selected at random. The high frequencies of high conservation values, combined with the low frequencies of low conservation values suggest that current PAs were selected efficiently. (b, c) Box-and-whisker plots for birds (b) and habitats (c) showing the proportion of the total distribution of each group of feature types that is represented in the existing protected area network. A dotted line indicates the random expectation for the representation of each feature class based on the amount of protected area in Romania (~ 20% of land surface area). (TIF) [file pone.0251950.s001.tif]

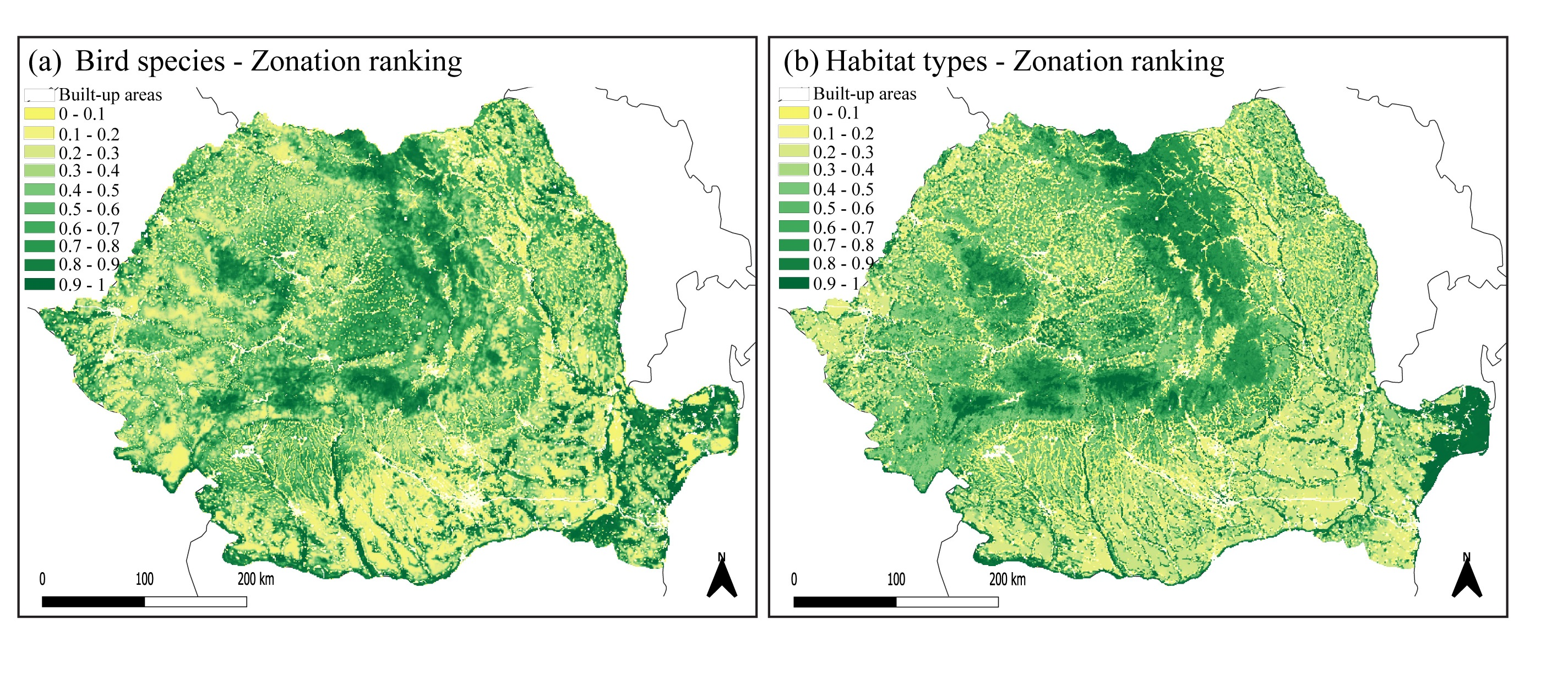

Supplement: S2 Fig — Study region with Zonation ranking based on (a) Bird species and (b) habitat types. Colors indicate importance ranking scores for conservation, with 0 meaning lowest importance and 1 meaning highest importance. Built-up areas are indicated in white and were excluded from prioritization. Figures are created by the authors. (TIF) [file pone.0251950.s002.tif]

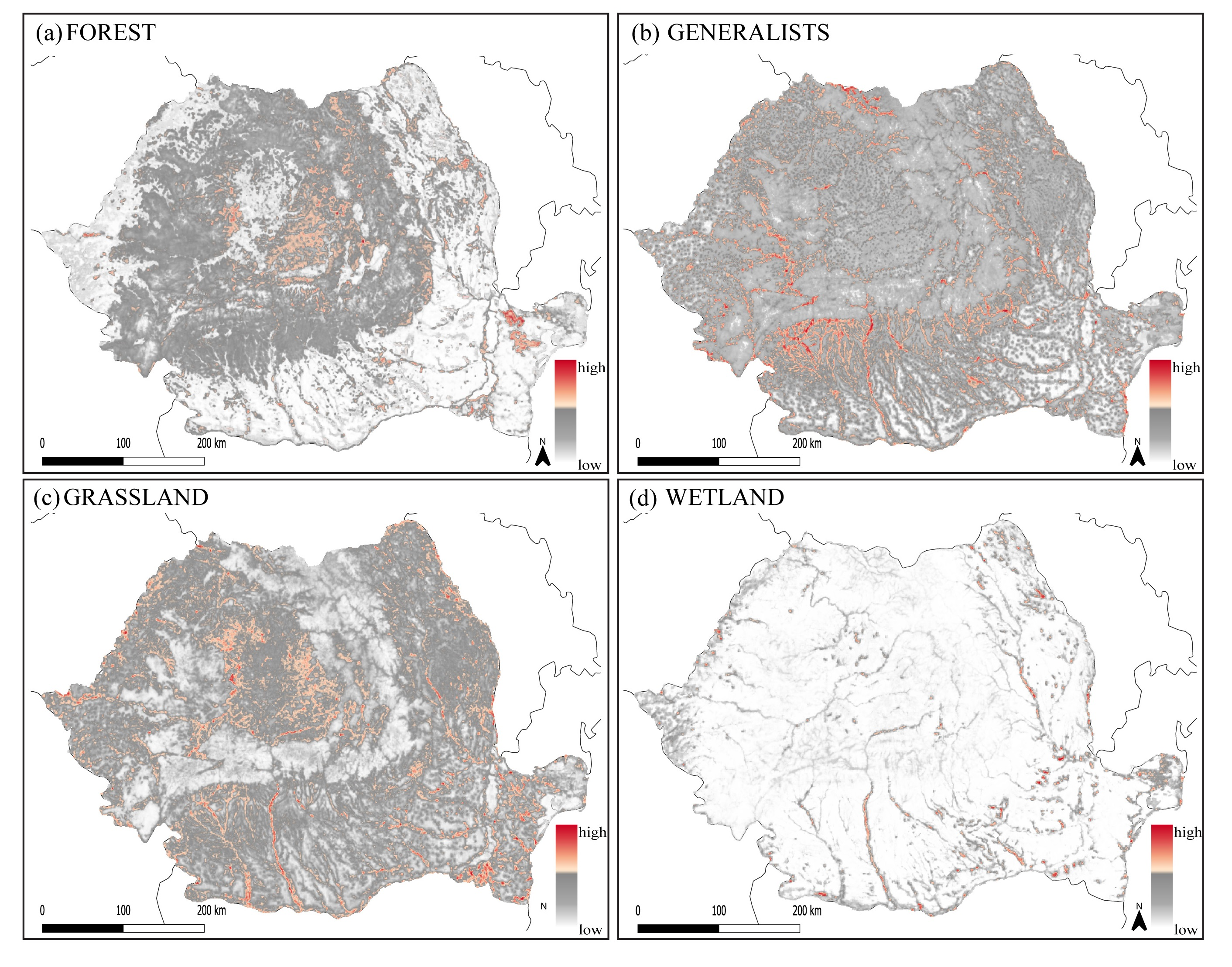

Supplement: S3 Fig — Overlapping bird species occurrences per breeding habitat group: (a) forests to (dense) woodland, (b) generalist and close to humans, (c) arable land, open woodland to grasslands, and (d) wetlands and shores. Red indicates species-rich areas; white to grey indicate no or low overlap of species occurrences. Figures are created by the authors. (TIF) [file pone.0251950.s003.tif]

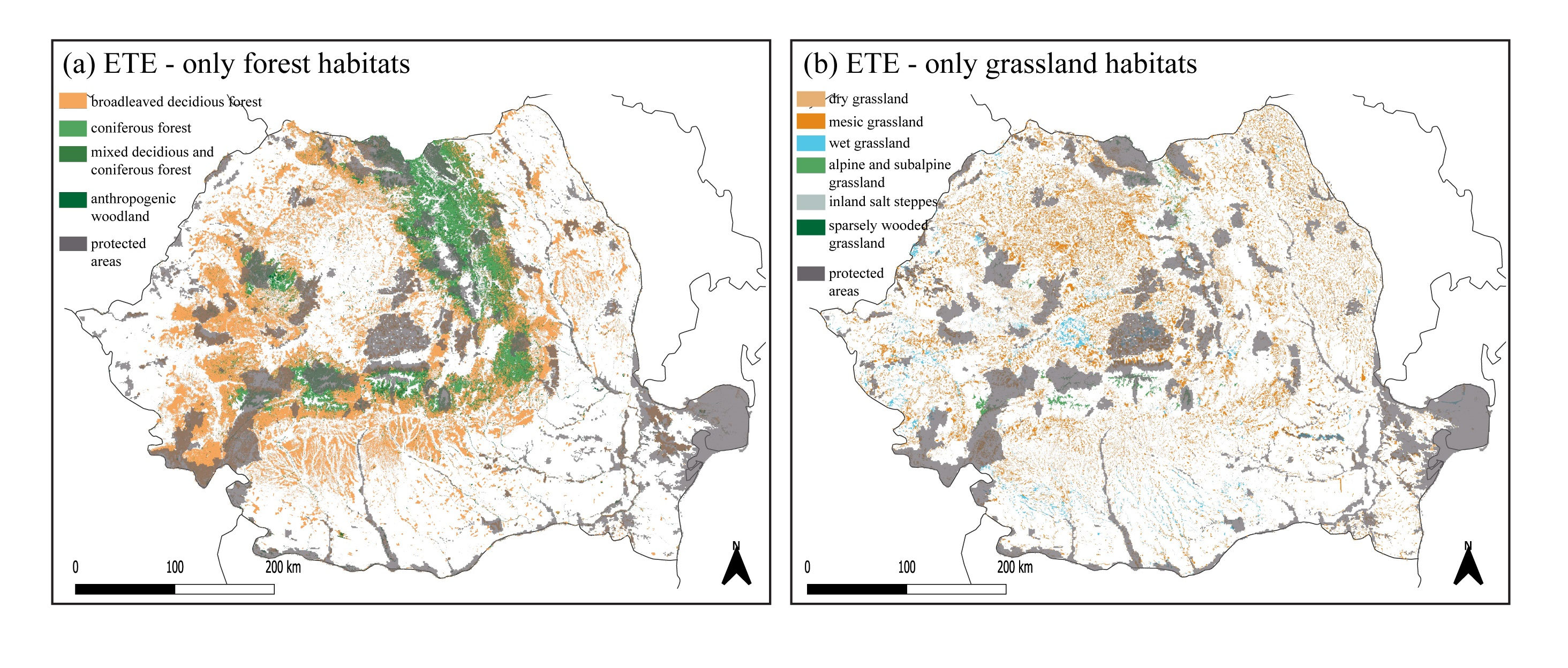

Supplement: S4 Fig — Study region with (a) forest habitats and (b) grassland habitats highlighted. The used protected area network is highlighted in grey. Figures are created by the authors. (TIF) [file pone.0251950.s004.tif]
