# Supplementary material for: Limited reciprocal surrogacy of bird and habitat diversity and inconsistencies in their representation in Romanian protected areas
Source: PLoS One. 2022 Feb 11;17(2):e0251950. doi: 10.1371/journal.pone.0251950 (PMC8836316; doi:10.1371/journal.pone.0251950)
Supplement: S1 Table — For each species we report the number of presence records, two estimates of model performance (AUC and omission error), the modelled range size, the threshold selected by an expert panel to omit areas where the species is considered absent with high certainty, the resolution of the final species distribution maps, and the regularization multiplier for each species in order to reduce the model complexity. (DOCX) [file pone.0251950.s005.docx]

**S1 Table** Summary of species distribution modelling (SDM) results from the upcoming Romanian Breeding Bird Atlas ((1),in preparation), a program run by Milvus Group Association and the Romanian Ornithological Society. For each species we report the number of presence records, two estimates of model performance (AUC and omission error), the modelled range size, the threshold selected by an expert panel to omit areas where the species is considered absent with high certainty, the resolution of the final species distribution maps, and the regularization multiplier for each species in order to reduce the model complexity.

| **species** | **common name** | **number of species presence locations** | **AUC** | **omission error** | **Distribution area (km2)** | **selected threshold** | **resolution** | **regularization multiplier** |
| --- | --- | --- | --- | --- | --- | --- | --- | --- |
| *Accipiter brevipes* | Levant sparrowhawk | 150 | 0.9764 | 0.04 | 11565 | MaxTrSenSpec | 1km | 1 |
| *Accipiter gentilis* | Northern goshawk | 999 | 0.7719 | 0.0761 | 149968 | Fcv5 | 1km | 1 |
| *Accipiter nisus* | Eurasian sparrowhawk | 2013 | 0.724 | 0.0586 | 142716 | Fcv5 | 1km | 1 |
| *Acrocephalus agricola* | Paddyfield warbler | 20 | 0.9991 | 0 | 324 | Fcv10 | 1km | 1 |
| *Acrocephalus arundinaceus* | Great reed warbler | 1565 | 0.8406 | 0.0798722 | 88117 | Fcv10 | 1km | 1 |
| *Acrocephalus palustris* | Marsh warbler | 1005 | 0.8781 | 0.03482587 | 95754 | Fcv10 | 1km | 1 |
| *Acrocephalus schoenobaenus* | Sedge warbler | 374 | 0.9522 | 0.04545455 | 36088 | EquEntThr | 1km | 1 |
| *Acrocephalus scirpaceus* | Eurasian reed warbler | 451 | 0.9592 | 0.12472648 | 12651 | P10TrPr | 1km | 1 |
| *Aegithalos caudatus* | Long-tailed tit | 1132 | 0.8498 | 0.00353357 | 137464 | BalTrOm | 1km | 1 |
| *Alauda arvensis* | Eurasian skylark | 3125 | 0.6891 | 0.04928 | 152366 | Fcv5 | 1km | 1 |
| *Alcedo atthis* | Common kingfisher | 498 | 0.9577 | 0.04696673 | 23341 | EquEntThr | 1km | 1 |
| *Anas platyrhynchos* | Mallard | 1128 | 0.8516 | 0.00353357 | 227900 | MinTrPr | 2km | 1 |
| *Anas strepera* | Gadwall | 232 | 0.9864 | 0.05172414 | 4420 | EquTrSenSpec | 2km | 1 |
| *Anser anser* | Greylag goose | 220 | 0.9868 | 0.05454546 | 4956 | EquTrSenSpec | 2km | 1 |
| *Anthus campestris* | Tawny pipit | 1006 | 0.8461 | 0.03280318 | 115121 | Fcv5 | 1km | 1 |
| *Anthus spinoletta* | Water pipit | 119 | 0.9934 | 0.00840336 | 4258 | Fcv5 | 1km | 1 |
| *Anthus trivialis* | Tree pipit | 908 | 0.8566 | 0.0154185 | 136779 | Fcv5 | 1km | 2 |
| *Aquila pomarina* | Lesser spotted eagle | 2202 | 0.7993 | 0.0999 | 35806 | P10TrPr | 1km | 1 |
| *Asio otus* | Long-eared owl | 999 | 0.815 | 0.00925926 | 168004 | BalTrOm | 1km | 1 |
| *Athene noctua* | Little owl | 2277 | 0.8041 | 0.03065356 | 127881 | Fcv5 | 1km | 1 |
| *Bonasa bonasia* | Hazel grouse | 201 | 0.9697 | 0.00588235 | 46795 | MinTrPr | 1km | 1 |
| *Bubo bubo* | Eurasian eagle-owl | 57 | 0.9712 | 0.04545455 | 10301 | EquTrSenSpec | 1km | 1 |
| *Burhinus oedicnemus* | Eurasian stone-curlew | 250 | 0.974 | 0.0326087 | 13795 | EquEntThr | 1km | 1 |
| *Buteo buteo* | Common buzzard | 8550 | 0.5589 | 0.0978 | 171619 | Fcv5 | 1km | 1 |
| *Buteo rufinus* | Long-legged buzzard | 820 | 0.8853 | 0.1 | 46687 | P10TrPr | 1km | 1 |
| *Calandrella brachydactyla* | Greater short-toed lark | 236 | 0.9802 | 0 | 22909 | Fcv5 | 1km | 1 |
| *Caprimulgus europaeus* | European nightjar | 452 | 0.8827 | 0.09398496 | 57247 | P10TrPr | 1km | 1 |
| *Carduelis cannabina* | Common linnet | 818 | 0.8548 | 0.03056235 | 115310 | EquEntThr | 1km | 1 |
| *Carduelis carduelis* | European goldfinch | 2319 | 0.74 | 0.03234153 | 170959 | Fcv5 | 1km | 1 |
| *Carduelis chloris* | European greenfinch | 1519 | 0.7858 | 0.04344964 | 143387 | EquEntThr | 1km | 1 |
| *Carduelis spinus* | Eurasian siskin | 74 | 0.9945 | 0 | 12725 | Fcv5 | 1km | 1 |
| *Certhia brachydactyla* | Short-toed treecreeper | 77 | 0.9945 | 0 | 7710 | Fcv10 | 1km | 1 |
| *Certhia familiaris* | Eurasian treecreeper | 823 | 0.89 | 0.00243013 | 118903 | BalTrOm | 1km | 1 |
| *Charadrius alexandrinus* | Kentish plover | 45 | 0.9979 | 0.02222222 | 796 | EquTrSenSpec | 2km | 1 |
| *Charadrius dubius* | Little ringed plover | 361 | 0.952 | 0.06371191 | 34244 | EquEntThr | 1km | 1 |
| *Cinclus cinclus* | White-throated dipper | 164 | 0.9809 | 0 | 36877 | MinTrPr | 1km | 2 |
| *Coccothraustes coccothraustes* | Hawfinch | 1324 | 0.8398 | 0.03096677 | 97413 | Fcv5 | 1km | 1 |
| *Columba livia domestica* | Domestic pigeon | 1924 | 0.8087 | 0.05405405 | 118003 | EquEntThr | 1km | 1 |
| *Columba oenas* | Stock dove | 424 | 0.9505 | 0.01886793 | 53847 | EquEntThr | 1km | 1 |
| *Coracias garrulus* | European roller | 743 | 0.9189 | 0.04845222 | 53189 | Fcv10 | 1km | 1 |
| *Coturnix coturnix* | Common quail | 2548 | 0.7158 | 0.04384966 | 150804 | Fcv5 | 1km | 1 |
| *Crex crex* | Corn crake | 2001 | 0.8026 | 0.05188199 | 102189 | Fcv10 | 1km | 1 |
| *Cuculus canorus* | Common cuckoo | 3570 | 0.6274 | 0 | 239633 | MinTrPr | 1km | 1 |
| *Cygnus olor* | Mute swan | 661 | 0.9505 | 0.13615734 | 14124 | EquTrSenSpec | 2km | 1 |
| *Delichon urbicum* | Common house martin | 1574 | 0.8265 | 0.1162643 | 84076 | P10TrPr | 1km | 1 |
| *Dendrocopos leucotos* | White-backed woodpecker | 454 | 0.9334 | 0.02777778 | 46683 | Fcv10 | 1km | 1 |
| *Dendrocopos major* | Great spotted woodpecker | 3122 | 0.7156 | 0.00348028 | 156345 | BalTrOm | 1km | 1 |
| *Dendrocopos medius* | Middle spotted woodpecker | 1209 | 0.8794 | 0.06470588 | 62628 | Fcv10 | 1km | 1 |
| *Dendrocopos minor* | Lesser spotted woodpecker | 659 | 0.8845 | 0.05238829 | 70416 | Fcv10 | 1km | 1 |
| *Dendrocopos syriacus* | Syrian woodpecker | 803 | 0.8487 | 0.07005495 | 97882 | Fcv10 | 1km | 1 |
| *Dryocopus martius* | Black woodpecker | 1785 | 0.8128 | 0.01915456 | 113305 | Fcv5 | 1km | 1 |
| *Emberiza cia* | Rock bunting | 112 | 0.9906 | 0 | 8507 | EquEntThr | 1km | 1 |
| *Emberiza cirlus* | Cirl bunting | 51 | 0.9964 | 0 | 6478 | Fcv10 | 1km | 1 |
| *Emberiza citrinella* | Yellowhammer | 1723 | 0.7891 | 0.01625073 | 138735 | Fcv5 | 1km | 1 |
| *Emberiza hortulana* | Ortolan bunting | 904 | 0.869 | 0.0199115 | 109369 | Fcv5 | 1km | 1 |
| *Emberiza melanocephala* | Black-headed bunting | 361 | 0.9601 | 0.00831025 | 46521 | Fcv5 | 1km | 1 |
| *Emberiza schoeniclus* | Common reed bunting | 325 | 0.9676 | 0.08307692 | 15601 | MaxTrSenSpec | 1km | 1 |
| *Erithacus rubecula* | European robin | 2414 | 0.7564 | 0.03769677 | 122279 | Fcv5 | 1km | 1 |
| *Falco subbuteo* | Eurasian hobby | 1486 | 0.7571 | 0.03376206 | 169871 | Fcv5 | 1km | 1 |
| *Falco tinnunculus* | Common kestrel | 5731 | 0.636 | 0.04747848 | 178879 | Fcv5 | 1km | 1 |
| *Ficedula parva* | Red-breasted flycatcher | 221 | 0.9718 | 0.0361991 | 27305 | EquEntThr | 1km | 1 |
| *Ficedula semitorquata* | Semicollared flycatcher | 12 | 0.9994 | 0 | 820 | EquEntThr | 1km | 1 |
| *Fringilla coelebs* | Common chaffinch | 3360 | 0.705 | 0.00565476 | 157344 | BalTrOm | 1km | 1 |
| *Fulica atra* | Eurasian coot | 719 | 0.9341 | 0.10292072 | 24588 | MaxTrSenSpec | 1km | 1 |
| *Galerida cristata* | Crested lark | 1099 | 0.8743 | 0.05732484 | 83983 | Fcv10 | 1km | 1 |
| *Gallinula chloropus* | Common moorhen | 736 | 0.9264 | 0.124 | 27176 | P10TrPr | 1km | 1 |
| *Garrulus glandarius* | Eurasian jay | 2100 | 0.7546 | 0.00619048 | 153409 | BalTrOm | 1km | 1 |
| *Hieraaetus pennatus* | Booted eagle | 342 | 0.9238 | 0.1696 | 12151 | MaxTrSenSpec | 1km | 1 |
| *Himantopus himantopus* | Black-winged stilt | 205 | 0.9751 | 0.07804878 | 15816 | MaxTrSenSpec | 2km | 1 |
| *Hippolais icterina* | Icterine warbler | 110 | 0.9903 | 0 | 24219 | MinTrPr | 1km | 1 |
| *Hippolais pallida* | Eastern olivaceous warbler | 55 | 0.997 | 0 | 3623 | Fcv5 | 1km | 1 |
| *Hirundo rustica* | Barn swallow | 3392 | 0.6856 | 0.04274764 | 175909 | Fcv5 | 1km | 1 |
| *Ixobrychus minutus* | Little bittern | 495 | 0.9527 | 0.094 | 21225 | MaxTrSenSpec | 1km | 1 |
| *Lanius collurio* | Red-backed shrike | 2413 | 0.6841 | 0.01533361 | 184748 | EquEntThr | 1km | 2 |
| *Lanius minor* | Lesser grey shrike | 1002 | 0.845 | 0.05189621 | 105810 | EquEntThr | 1km | 1 |
| *Locustella fluviatilis* | River warbler | 225 | 0.9763 | 0 | 30695 | EquEntThr | 1km | 1 |
| *Locustella luscinioides* | Savi's warbler | 538 | 0.9438 | 0.0464684 | 38171 | EquEntThr | 1km | 1 |
| *Loxia curvirostra* | Red crossbill | 465 | 0.954 | 0.03655914 | 28401 | Fcv5 | 1km | 2 |
| *Lullula arborea* | Woodlark | 1348 | 0.8227 | 0.05267062 | 100524 | Fcv10 | 1km | 1 |
| *Luscinia luscinia* | Thrush nightingale | 433 | 0.9387 | 0.0300231 | 34492 | MaxTrSenSpec | 1km | 1 |
| *Luscinia megarhynchos* | Common nightingale | 1810 | 0.8172 | 0.02541437 | 102270 | Fcv5 | 1km | 1 |
| *Melanocorypha calandra* | Calandra lark | 370 | 0.9669 | 0.01891892 | 21027 | Fcv10 | 1km | 1 |
| *Merops apiaster* | European bee-eater | 1057 | 0.8285 | 0.05014191 | 113904 | Fcv10 | 1km | 1 |
| *Motacilla alba* | White wagtail | 2190 | 0.7405 | 0.06347032 | 154479 | Fcv10 | 1km | 1 |
| *Motacilla cinerea* | Grey wagtail | 639 | 0.9275 | 0.02816901 | 58774 | Fcv5 | 1km | 1 |
| *Motacilla flava* | Western yellow wagtail | 1537 | 0.8085 | 0.0540013 | 117467 | Fcv5 | 1km | 1 |
| *Nucifraga caryocatactes* | Spotted nutcracker | 187 | 0.9838 | 0.00534759 | 22875 | Fcv5 | 1km | 1 |
| *Oenanthe isabellina* | Isabelline wheatear | 176 | 0.9894 | 0.01136364 | 5426 | Fcv5 | 1km | 1 |
| *Oenanthe oenanthe* | Northern wheatear | 782 | 0.8721 | 0.04347826 | 111022 | Fcv10 | 1km | 1 |
| *Oriolus oriolus* | Eurasian golden oriole | 2213 | 0.7191 | 0.01897876 | 167525 | Fcv5 | 1km | 1 |
| *Otus scops* | Eurasian scops owl | 1788 | 0.8031 | 0.05876494 | 105413 | Fcv10 | 1km | 1 |
| *Parus caeruleus* | Eurasian blue tit | 1634 | 0.774 | 0.06670747 | 119629 | Fcv10 | 1km | 1 |
| *Parus cristatus* | European crested tit | 449 | 0.9618 | 0.02004454 | 22003 | Fcv5 | 1km | 1 |
| *Parus lugubris* | Sombre tit | 117 | 0.9907 | 0 | 18273 | Fcv10 | 1km | 1 |
| *Parus major* | Great tit | 3969 | 0.6469 | 0.0047871 | 206798 | BalTrOm | 1km | 1 |
| *Parus montanus* | Willow tit | 592 | 0.9471 | 0 | 37331 | MinTrPr | 1km | 1 |
| *Parus palustris* | Marsh tit | 1492 | 0.8079 | 0.08176944 | 92651 | Fcv10 | 1km | 1 |
| *Passer domesticus* | House sparrow | 3374 | 0.7389 | 0.09780676 | 116015 | Fcv10 | 1km | 1 |
| *Passer hispaniolensis* | Spanish sparrow | 464 | 0.9565 | 0.00431035 | 42447 | Fcv5 | 1km | 1 |
| *Phoenicurus ochruros* | Black redstart | 1332 | 0.8471 | 0.1036036 | 76824 | P10TrPr | 1km | 1 |
| *Phylloscopus collybita* | Common chiffchaff | 2395 | 0.739 | 0.03256785 | 129632 | Fcv5 | 1km | 1 |
| *Phylloscopus sibilatrix* | Wood warbler | 486 | 0.9353 | 0.00205761 | 88040 | Fcv5 | 1km | 1 |
| *Pica pica* | Eurasian magpie | 4186 | 0.6655 | 0.00716675 | 198219 | BalTrOm | 1km | 1 |
| *Picoides tridactylus* | Eurasian three-toed woodpecker | 142 | 0.9795 | 0.01526718 | 18665 | Fcv5 | 1km | 1 |
| *Picus canus* | Grey-headed woodpecker | 1655 | 0.8156 | 0.05707763 | 97447 | Fcv10 | 1km | 1 |
| *Picus viridis* | European green woodpecker | 1762 | 0.8221 | 0.06904401 | 87314 | Fcv10 | 1km | 1 |
| *Podiceps cristatus* | Great crested grebe | 645 | 0.9511 | 0.13178295 | 12888 | P10TrPr | 2km | 1 |
| *Prunella collaris* | Alpine accentor | 35 | 0.9982 | 0 | 641 | Fcv10 | 1km | 1 |
| *Prunella modularis* | Dunnock | 256 | 0.9777 | 0.0078125 | 24960 | MinTrPr | 1km | 1 |
| *Pyrrhula pyrrhula* | Eurasian bullfinch | 250 | 0.9745 | 0.008 | 24755 | Fcv5 | 1km | 1 |
| *Rallus aquaticus* | Water rail | 234 | 0.9771 | 0.08016878 | 12258 | EquTrSenSpec | 1km | 1 |
| *Recurvirostra avosetta* | Pied avocet | 103 | 0.9897 | 0.04807692 | 7332 | MaxTrSenSpec | 2km | 1 |
| *Regulus regulus* | Goldcrest | 463 | 0.9562 | 0 | 44825 | Fcv1 | 1km | 1 |
| *Saxicola rubetra* | Whinchat | 496 | 0.9099 | 0.05645161 | 69063 | Fcv10 | 1km | 1 |
| *Saxicola torquatus* | African stonechat | 1126 | 0.8449 | 0.04618117 | 100266 | Fcv10 | 1km | 1 |
| *Serinus serinus* | European serin | 457 | 0.952 | 0.10065646 | 21589 | MaxTrSenSpec | 1km | 1 |
| *Sitta europaea* | Eurasian nuthatch | 2121 | 0.7594 | 0.04196134 | 125216 | Fcv5 | 1km | 1 |
| *Streptopelia decaocto* | Eurasian collared dove | 3100 | 0.776 | 0.03870968 | 126654 | Fcv5 | 1km | 1 |
| *Streptopelia turtur* | European turtle dove | 1451 | 0.7861 | 0.05237767 | 128254 | EquEntThr | 1km | 1 |
| *Strix aluco* | Tawny owl | 1811 | 0.8131 | 0.02822926 | 126119 | Fcv5 | 1km | 1 |
| *Strix uralensis* | Ural owl | 1122 | 0.8849 | 0.00621118 | 92410 | Fcv5 | 1km | 1 |
| *Sturnus vulgaris* | Common starling | 2691 | 0.6964 | 0.0040877 | 204886 | BalTrOm | 1km | 1 |
| *Sylvia atricapilla* | Eurasian blackcap | 2408 | 0.7241 | 0.01204319 | 149946 | EquEntThr | 1km | 2 |
| *Sylvia communis* | Common whitethroat | 1967 | 0.7417 | 0.03202847 | 163133 | Fcv5 | 1km | 2 |
| *Sylvia curruca* | Lesser whitethroat | 1369 | 0.7813 | 0.01826151 | 179018 | Fcv5 | 1km | 1 |
| *Sylvia nisoria* | Barred warbler | 349 | 0.9023 | 0.12320917 | 59416 | P10TrPr | 1km | 2 |
| *Tachybaptus ruficollis* | Little grebe | 328 | 0.9665 | 0.15243902 | 9976 | P10TrPr | 2km | 1 |
| *Tadorna ferruginea* | Ruddy shelduck | 85 | 0.9959 | 0 | 4220 | Fcv10 | 2km | 1 |
| *Tadorna tadorna* | Common shelduck | 144 | 0.9931 | 0.00694444 | 2512 | MaxTrSenSpec | 2km | 1 |
| *Troglodytes troglodytes* | Eurasian wren | 897 | 0.8818 | 0.02564103 | 85219 | Fcv5 | 1km | 1 |
| *Turdus merula* | Common blackbird | 3046 | 0.7114 | 0.03184504 | 136404 | Fcv5 | 1km | 1 |
| *Turdus philomelos* | Song thrush | 2471 | 0.7402 | 0.02832861 | 130876 | Fcv5 | 1km | 1 |
| *Turdus torquatus* | Ring ouzel | 109 | 0.9933 | 0 | 9846 | Fcv5 | 1km | 1 |
| *Turdus viscivorus* | Mistle thrush | 803 | 0.8631 | 0.00871731 | 113118 | BalTrOm | 1km | 2 |
| *Tyto alba* | Western barn owl | 148 | 0.9651 | 0.05185185 | 14086 | MaxTrSenSpec | 1km | 1 |
| *Upupa epops* | Eurasian hoopoe | 1680 | 0.7692 | 0.0702381 | 128793 | Fcv10 | 1km | 1 |
| *Vanellus vanellus* | Northern lapwing | 993 | 0.8646 | 0.05840886 | 24204 | Fcv10 | 2km | 1 |

*threshold abbreviations: BalTrOm (balance training omission, predicted area, and threshold value), EquEntThr (equate entropy of thresholded and original distributions), EquTrSenSpec (equal training sensitivity and specificity), Fcv1 (fixed cumulative value 1), Fcv5 (fixed cumulative value 5), Fcv10 (fixed cumulative value 10), MaxTrSenSpec (maximum training sensitivity and specificity), MinTrPr (minimum training presence), P10TrPr (10^th^ percentile training presence)

1. Fântânâ C, Kovács I. The Romanian breeding bird atlas 2006-2017, a common scheme of Milvus Group Association and the Romanian Ornithological Society. in preparation. 2020.
