# Supplementary material for: Limited reciprocal surrogacy of bird and habitat diversity and inconsistencies in their representation in Romanian protected areas
Source: PLoS One. 2022 Feb 11;17(2):e0251950. doi: 10.1371/journal.pone.0251950 (PMC8836316; doi:10.1371/journal.pone.0251950)
Supplement: S2 Table — Variables marked in bold were included in the models, after highly cross-correlated variables (with Pearson correlation coefficient > 0.8) were omitted. (DOCX) [file pone.0251950.s006.docx]

**S2 Table** Environmental variables used for species distribution modeling for the Romanian Breeding Bird Atlas ((1), in preparation). Variables marked in bold were included in the models, after highly cross-correlated variables (with Pearson correlation coefficient > 0.8) were omitted.

| **Code** | **Description** | **Source** |
| --- | --- | --- |
| alt | Altitude | CliMond repositories |
| Bio01 | Annual mean temperature (°C) | CliMond repositories |
| **Bio02** | **Mean diurnal temperature range (mean (period max-min)) (°C)** | **CliMond repositories** |
| Bio03 | Isothermality (Bio02 ÷ Bio07) | CliMond repositories |
| Bio04 | Temperature seasonality (coefficient of variation) | CliMond repositories |
| Bio05 | Max temperature of warmest week (°C) | CliMond repositories |
| Bio06 | Min temperature of coldest week (°C) | CliMond repositories |
| **Bio07** | **Temperature annual range (Bio05-Bio06) (°C)** | **CliMond repositories** |
| Bio08 | Mean temperature of wettest quarter (°C) | CliMond repositories |
| **Bio09** | **Mean temperature of driest quarter (°C)** | **CliMond repositories** |
| Bio10 | Mean temperature of warmest quarter (°C) | CliMond repositories |
| Bio11 | Mean temperature of coldest quarter (°C) | CliMond repositories |
| Bio12 | Annual precipitation (mm) | CliMond repositories |
| Bio13 | Precipitation of wettest week (mm) | CliMond repositories |
| Bio14 | Precipitation of driest week (mm) | CliMond repositories |
| **Bio15** | **Precipitation seasonality (coefficient of variation)** | **CliMond repositories** |
| Bio16 | Precipitation of wettest quarter (mm) | CliMond repositories |
| Bio17 | Precipitation of driest quarter (mm) | CliMond repositories |
| Bio18 | Precipitation of warmest quarter (mm) | CliMond repositories |
| **Bio19** | **Precipitation of coldest quarter (mm)** | **CliMond repositories** |
| **Bio20** | **Annual mean radiation (W m-2)** | **CliMond repositories** |
| Bio21 | Highest weekly radiation (W m-2) | CliMond repositories |
| Bio22 | Lowest weekly radiation (W m-2 | CliMond repositories |
| **Bio23** | **Radiation seasonality (coefficient of variation)** | **CliMond repositories** |
| **Bio24** | **Radiation of wettest quarter (W m-2)** | **CliMond repositories** |
| Bio25 | Radiation of driest quarter (W m-2) | CliMond repositories |
| Bio26 | Radiation of warmest quarter (W m-2) | CliMond repositories |
| **Bio27** | **Radiation of coldest quarter (W m-2)** | **CliMond repositories** |
| Bio28 | Annual mean moisture index | CliMond repositories |
| Bio29 | Highest weekly moisture index | CliMond repositories |
| Bio30 | Lowest weekly moisture index | CliMond repositories |
| Bio31 | Moisture index seasonality (C of V) | CliMond repositories |
| Bio32 | Mean moisture index of wettest quarter | CliMond repositories |
| Bio33 | Mean moisture index of driest quarter | CliMond repositories |
| Bio34 | Mean moisture index of warmest quarter | CliMond repositories |
| Bio35 | Mean moisture index of coldest quarter | CliMond repositories |
| **Bio36** | **First principal component of the first 35 Bioclim variables** | **CliMond repositories** |
| **Bio37** | **Second principal component of the first 35 Bioclim variables** | **CliMond repositories** |
| Bio38 | Third principal component of the first 35 Bioclim variables | CliMond repositories |
| **Bio39** | **Fourth principal component of the first 35 Bioclim variables** | **CliMond repositories** |
| Bio40 | Fifth principal component of the first 35 Bioclim variables | CliMond repositories |
| aglim1 | Code of the most important limitation to agricultural use of the STU | European Soil Data Centre (ESDAC), esdac.jrc.ec.europa.eu, European Commission, Joint Research Centre |
| aglim2 | Code of a secondary limitation to agricultural use of the STU | European Soil Data Centre (ESDAC), esdac.jrc.ec.europa.eu, European Commission, Joint Research Centre |
| fao90fu | Full soil code of the STU from the 1990 FAO-UNESCO Soil Legend | European Soil Data Centre (ESDAC), esdac.jrc.ec.europa.eu, European Commission, Joint Research Centre |
| parmado | Code for dominant parent material of the STU | European Soil Data Centre (ESDAC), esdac.jrc.ec.europa.eu, European Commission, Joint Research Centre |
| parmase | Code for secondary parent material of the STU | European Soil Data Centre (ESDAC), esdac.jrc.ec.europa.eu, European Commission, Joint Research Centre |
| slopedo | Dominant slope class of the STU | European Soil Data Centre (ESDAC), esdac.jrc.ec.europa.eu, European Commission, Joint Research Centre |
| slopese | Secondary slope class of the STU | European Soil Data Centre (ESDAC), esdac.jrc.ec.europa.eu, European Commission, Joint Research Centre |
| usedo | Code for dominant land use of the STU | European Soil Data Centre (ESDAC), esdac.jrc.ec.europa.eu, European Commission, Joint Research Centre |
| usese | Code for secondary land use of the STU | European Soil Data Centre (ESDAC), esdac.jrc.ec.europa.eu, European Commission, Joint Research Centre |
| vs | Volume of stones | European Soil Data Centre (ESDAC), esdac.jrc.ec.europa.eu, European Commission, Joint Research Centre |
| wm1 | Code for normal presence and purpose of an existing water management system in agricultural land on more than 50% of the STU | European Soil Data Centre (ESDAC), esdac.jrc.ec.europa.eu, European Commission, Joint Research Centre |
| wr | Dominant annual average soil water regime class of the soil profile of the STU | European Soil Data Centre (ESDAC), esdac.jrc.ec.europa.eu, European Commission, Joint Research Centre |
| il | Code for the presence of an impermeable layer within the soil profile of the STU | European Soil Data Centre (ESDAC), esdac.jrc.ec.europa.eu, European Commission, Joint Research Centre |
| pmh | Parent material hydrogeological type | European Soil Data Centre (ESDAC), esdac.jrc.ec.europa.eu, European Commission, Joint Research Centre |
| dr | Depth to rock | European Soil Data Centre (ESDAC), esdac.jrc.ec.europa.eu, European Commission, Joint Research Centre |
| **ILSWE** | **Index of Land Susceptibility to Wind Erosion 1981-2010** | **European Soil Data Centre (ESDAC), esdac.jrc.ec.europa.eu, European Commission, Joint Research Centre** |
| desiccation | Desiccation | European Soil Data Centre (ESDAC), esdac.jrc.ec.europa.eu, European Commission, Joint Research Centre |
| andosol | Soil type extracted from the 1990 FAO-UNESCO Soil Legend | European Soil Data Centre (ESDAC), esdac.jrc.ec.europa.eu, European Commission, Joint Research Centre |
| arenosol | Soil type extracted from the 1990 FAO-UNESCO Soil Legend | European Soil Data Centre (ESDAC), esdac.jrc.ec.europa.eu, European Commission, Joint Research Centre |
| cambisol | Soil type extracted from the 1990 FAO-UNESCO Soil Legend | European Soil Data Centre (ESDAC), esdac.jrc.ec.europa.eu, European Commission, Joint Research Centre |
| chernozem | Soil type extracted from the 1990 FAO-UNESCO Soil Legend | European Soil Data Centre (ESDAC), esdac.jrc.ec.europa.eu, European Commission, Joint Research Centre |
| fluvisol | Soil type extracted from the 1990 FAO-UNESCO Soil Legend | European Soil Data Centre (ESDAC), esdac.jrc.ec.europa.eu, European Commission, Joint Research Centre |
| gleysol | Soil type extracted from the 1990 FAO-UNESCO Soil Legend | European Soil Data Centre (ESDAC), esdac.jrc.ec.europa.eu, European Commission, Joint Research Centre |
| greyzem | Soil type extracted from the 1990 FAO-UNESCO Soil Legend | European Soil Data Centre (ESDAC), esdac.jrc.ec.europa.eu, European Commission, Joint Research Centre |
| kastanozem | Soil type extracted from the 1990 FAO-UNESCO Soil Legend | European Soil Data Centre (ESDAC), esdac.jrc.ec.europa.eu, European Commission, Joint Research Centre |
| leptosol | Soil type extracted from the 1990 FAO-UNESCO Soil Legend | European Soil Data Centre (ESDAC), esdac.jrc.ec.europa.eu, European Commission, Joint Research Centre |
| luvisol | Soil type extracted from the 1990 FAO-UNESCO Soil Legend | European Soil Data Centre (ESDAC), esdac.jrc.ec.europa.eu, European Commission, Joint Research Centre |
| phaeozem | Soil type extracted from the 1990 FAO-UNESCO Soil Legend | European Soil Data Centre (ESDAC), esdac.jrc.ec.europa.eu, European Commission, Joint Research Centre |
| podzol | Soil type extracted from the 1990 FAO-UNESCO Soil Legend | European Soil Data Centre (ESDAC), esdac.jrc.ec.europa.eu, European Commission, Joint Research Centre |
| regosol | Soil type extracted from the 1990 FAO-UNESCO Soil Legend | European Soil Data Centre (ESDAC), esdac.jrc.ec.europa.eu, European Commission, Joint Research Centre |
| vertisol | Soil type extracted from the 1990 FAO-UNESCO Soil Legend | European Soil Data Centre (ESDAC), esdac.jrc.ec.europa.eu, European Commission, Joint Research Centre |
| **dist2water1km** | **Distance to water** | **Covariate** **derivative from Corine Land Cover - compiled and calculated by SOVON (Netherlands)** |
| div3_1_10 | Forest in surrounding area of x km | Covariate derivative from Corine Land Cover - compiled and calculated by SOVON (Netherlands) |
| **div3_1_100** | **Forest in surrounding area of x km** | **Covariate** **derivative from Corine Land Cover - compiled and calculated by SOVON (Netherlands)** |
| div3_1_20 | Forest in surrounding area of x km | Covariate derivative from Corine Land Cover - compiled and calculated by SOVON (Netherlands) |
| div3_1_50 | Forest in surrounding area of x km | Covariate derivative from Corine Land Cover - compiled and calculated by SOVON (Netherlands) |
| **div3_2_10** | **shrub and/or herbaceous vegetation in surrounding area of x km** | **Covariate derivative from Corine Land Cover - compiled and calculated by SOVON (Netherlands)** |
| div3_2_100 | shrub and/or herbaceous vegetation in surrounding area of x km | Covariate derivative from Corine Land Cover - compiled and calculated by SOVON (Netherlands) |
| div3_2_20 | shrub and/or herbaceous vegetation in surrounding area of x km | Covariate derivative from Corine Land Cover - compiled and calculated by SOVON (Netherlands) |
| **div3_2_50** | **shrub and/or herbaceous vegetation in surrounding area of x km** | **Covariate derivative from Corine Land Cover - compiled and calculated by SOVON (Netherlands)** |
| **div3_3_10** | **open spaces with little or no vegetation** | **Covariate derivative from Corine Land Cover - compiled and calculated by SOVON (Netherlands)** |
| div3_3_100 | open spaces with little or no vegetation | Covariate derivative from Corine Land Cover - compiled and calculated by SOVON (Netherlands) |
| div3_3_20 | open spaces with little or no vegetation | Covariate derivative from Corine Land Cover - compiled and calculated by SOVON (Netherlands) |
| **div3_3_50** | **open spaces with little or no vegetation** | **Covariate derivative from Corine Land Cover - compiled and calculated by SOVON (Netherlands)** |
| div4_1_10 | inland wetlands in surrounding area of x km | Covariate derivative from Corine Land Cover - compiled and calculated by SOVON (Netherlands) |
| div4_1_100 | inland wetlands in surrounding area of x km | Covariate derivative from Corine Land Cover - compiled and calculated by SOVON (Netherlands) |
| div4_1_20 | inland wetlands in surrounding area of x km | Covariate derivative from Corine Land Cover - compiled and calculated by SOVON (Netherlands) |
| **div4_1_50** | **inland wetlands in surrounding area of x km** | **Covariate derivative from Corine Land Cover - compiled and calculated by SOVON (Netherlands)** |
| div4_2_10 | coastal wetlands in surrounding area of x km | Covariate derivative from Corine Land Cover - compiled and calculated by SOVON (Netherlands) |
| div4_2_100 | coastal wetlands in surrounding area of x km | Covariate derivative from Corine Land Cover - compiled and calculated by SOVON (Netherlands) |
| div4_2_20 | coastal wetlands in surrounding area of x km | Covariate derivative from Corine Land Cover - compiled and calculated by SOVON (Netherlands) |
| **div4_2_50** | **coastal wetlands in surrounding area of x km** | **Covariate derivative from Corine Land Cover - compiled and calculated by SOVON (Netherlands)** |
| div5_1_10 | inland waters in surrounding area of x km | Covariate derivative from Corine Land Cover - compiled and calculated by SOVON (Netherlands) |
| **div5_1_100** | **inland waters in surrounding area of x km** | **Covariate derivative from Corine Land Cover - compiled and calculated by SOVON (Netherlands)** |
| **div5_1_20** | **inland waters in surrounding area of x km** | **Covariate derivative from Corine Land Cover - compiled and calculated by SOVON (Netherlands)** |
| div5_1_50 | inland waters in surrounding area of x km | Covariate derivative from Corine Land Cover - compiled and calculated by SOVON (Netherlands) |
| **eco_f0** | **Littoral rock and other hard substrates** | **EUNIS: Ecosystem types of Europe** |
| **eco_f10** | **Coastal lagoons** | **EUNIS: Ecosystem types of Europe** |
| **eco_f11** | **Coastal dunes and sandy shores** | **EUNIS: Ecosystem types of Europe** |
| **eco_f14** | **Surface standing waters** | **EUNIS: Ecosystem types of Europe** |
| **eco_f15** | **Surface running waters** | **EUNIS: Ecosystem types of Europe** |
| **eco_f16** | **Littoral zone of inland surface waterbodies** | **EUNIS: Ecosystem types of Europe** |
| **eco_f20** | **Base-rich fens and calcareous spring mires** | **EUNIS: Ecosystem types of Europe** |
| **eco_f21** | **Sedge and reedbeds, normally without free-standing water** | **EUNIS: Ecosystem types of Europe** |
| **eco_f23** | **Dry grasslands** | **EUNIS: Ecosystem types of Europe** |
| **eco_f24** | **Mesic grasslands** | **EUNIS: Ecosystem types of Europe** |
| **eco_f25** | **Seasonally wet and wet grasslands** | **EUNIS: Ecosystem types of Europe** |
| **eco_f26** | **Alpine and subalpine grasslands** | **EUNIS: Ecosystem types of Europe** |
| **eco_f28** | **Inland salt steppes** | **EUNIS: Ecosystem types of Europe** |
| **eco_f31** | **Arctic, alpine and subalpine scrub** | **EUNIS: Ecosystem types of Europe** |
| **eco_f32** | **Temperate and Mediterranean-montane scrub** | **EUNIS: Ecosystem types of Europe** |
| eco_f38 | Riverine and fen scrubs | EUNIS: Ecosystem types of Europe |
| **eco_f40** | **Shrub plantations** | **EUNIS: Ecosystem types of Europe** |
| **eco_f41** | **Broadleaved deciduous woodland** | **EUNIS: Ecosystem types of Europe** |
| **eco_f43** | **Coniferous woodland** | **EUNIS: Ecosystem types of Europe** |
| **eco_f44** | **Mixed deciduous and coniferous woodland** | **EUNIS: Ecosystem types of Europe** |
| **eco_f45** | **Lines of trees, small anthropogenic woodlands, recently felled woodland, early-stage woodland and coppice** | **EUNIS: Ecosystem types of Europe** |
| **eco_f47** | **Screes** | **EUNIS: Ecosystem types of Europe** |
| **eco_f48** | **Inland cliffs, rock pavements and outcrops** | **EUNIS: Ecosystem types of Europe** |
| **eco_f50** | **Miscellaneous inland habitats with very sparse or no vegetation** | **EUNIS: Ecosystem types of Europe** |
| **eco_f52** | **Arable land and market gardens** | **EUNIS: Ecosystem types of Europe** |
| **eco_f53** | **Cultivated areas of gardens and parks** | **EUNIS: Ecosystem types of Europe** |
| **eco_f54** | **Buildings of cities, towns and villages** | **EUNIS: Ecosystem types of Europe** |
| **eco_f55** | **Low density buildings** | **EUNIS: Ecosystem types of Europe** |
| **eco_f56** | **Extractive industrial sites** | **EUNIS: Ecosystem types of Europe** |
| **eco_f57** | **Transport networks and other constructed hard-surfaced areas** | **EUNIS: Ecosystem types of Europe** |
| **eco_f59** | **Waste deposits** | **EUNIS: Ecosystem types of Europe** |
| **Ecoreg** | **Map of relief units** | **http://www.geo-spatial.org/download/harta-unitati-relief-romania** |
| fma_f2_1km | Forest management: close-to-nature |  |
| fma_f3_1km | Forest management: combined objective forestry |  |
| fma_f4_1km | Forest management: even-aged forestry |  |
| fma_f5_1km | Forest management: short rotation forestry |  |
| **soil_clay** | **Soil: clay content** | **European Soil Data Centre (ESDAC), esdac.jrc.ec.europa.eu, European Commission, Joint Research Centre** |
| **soil_oc** | **Soil: organic carbon content** | **European Soil Data Centre (ESDAC), esdac.jrc.ec.europa.eu, European Commission, Joint Research Centre** |
| **soil_ph** | **Soil: PH** | **European Soil Data Centre (ESDAC), esdac.jrc.ec.europa.eu, European Commission, Joint Research Centre** |
| **soil_salt** | **Soil: availability of salt** | **European Soil Data Centre (ESDAC), esdac.jrc.ec.europa.eu, European Commission, Joint Research Centre** |
| **soil_silt** | **Soil: silt content** | **European Soil Data Centre (ESDAC), esdac.jrc.ec.europa.eu, European Commission, Joint Research Centre** |
| **ndeposition_1km** | **Total nitrogen deposition** |  |
| sdeposition_1km | Total deposition of oxidized sulphur |  |
| **pet_he_yr** | **Potential Evapotranspiration** |  |
| tsum | Total sum of daily average temperatures |  |
| inhabited_land_clc | Localities, anthropized surfaces | CorineLandCover2006 |
| urban_habitat_clc | Urban habitats | CorineLandCover2006 |
| rural_habitat_clc | Rural habitats in the locality | CorineLandCover2006 |
| rural_landskape_clc | Rural landscape | CorineLandCover2006 |
| arable_clc | Ploughed land, intense irrigated agriculture | CorineLandCover2006 |
| open_habitat_clc | Open habitats, pastures | CorineLandCover2006 |
| shrubs_clc | Shrubland | CorineLandCover2006 |
| rock_clc | Stone and gravel surfaces | CorineLandCover2006 |
| wetland_clc | Wetlands | CorineLandCover2006 |
| water_body_clc | Water bodies | CorineLandCover2006 |
| conifer_forest_wwf | Temperate coniferous forest | Project LIFE05 NAT/RO/000176 "Habitate prioritare alpine, subalpine si forestiere din Romania" |
| fagus_coniferus_mix_wwf | Mixed deciduous and coniferous forest | Project LIFE05 NAT/RO/000176 "Habitate prioritare alpine, subalpine si forestiere din Romania" |
| fagus_forest_wwf | Beech forest | Project LIFE05 NAT/RO/000176 "Habitate prioritare alpine, subalpine si forestiere din Romania" |
| riparian_forest_wwf | Riparian forest | Project LIFE05 NAT/RO/000176 "Habitate prioritare alpine, subalpine si forestiere din Romania" |
| quercus_forest_wwf | Oak forest | Project LIFE05 NAT/RO/000176 "Habitate prioritare alpine, subalpine si forestiere din Romania" |
| **RO_population_2011** | **Human population estimate of Romania** | **ROStat** |
| **ALBH_2014apr** | **Bihemispheric Albedo** | **Copernicus Global Land Service** |
| **ALDH_2014apr** | **Directional Albedo** | **Copernicus Global Land Service** |
| **DMP_2016apr** | **Dry Matter Productivity** | **Copernicus Global Land Service** |
| FAPAR_2016apr | Fraction of Absorbed Photosynthetically Active Radiation | Copernicus Global Land Service |
| **FCOVER_2016apr** | **Fraction of green Vegetation Cover** | **Copernicus Global Land Service** |
| **LAI_2016apr** | **Leaf Area index** | **Copernicus Global Land Service** |
| **NDVI_2014apr** | **Normalized Difference Vegetation Index** | **Copernicus Global Land Service** |
| tcd_33 | Tree Cover Density 2012 0-33% coverage | Copernicus Global Land Service |
| tcd_33_66 | Tree Cover Density 2012 33-66% coverage | Copernicus Global Land Service |
| **tcd_66_100** | **Tree Cover Density 2012 66-100% coverage** | **Copernicus Global Land Service** |
| **tcd_total** | **Tree Cover Density 2012** | **Copernicus Global Land Service** |
| **TOCR_2014apr** | **Top Of Canopy Reflectance** | **Copernicus Global Land Service** |
| **VCI_2016apr** | **Vegetation Condition Index** | **Copernicus Global Land Service** |

1. Fântânâ C, Kovács I. The Romanian breeding bird atlas 2006-2017, a common scheme of Milvus Group Association and the Romanian Ornithological Society. in preparation. 2020.
