# Supplementary material for: Limited reciprocal surrogacy of bird and habitat diversity and inconsistencies in their representation in Romanian protected areas
Source: PLoS One. 2022 Feb 11;17(2):e0251950. doi: 10.1371/journal.pone.0251950 (PMC8836316; doi:10.1371/journal.pone.0251950)
Supplement: S3 Table — For each habitat type, the abbreviation, the habitat name, the geometric and thematic reliability values [61], range size and AUC of the Zonation performance curve are provided. Some habitat types were listed with several accuracy values, based on how often they were translated and used in the final EUNIS classification. We followed the ‘combined reliabilities’ approach [61] and took the maximum reliability value as a reference value (indicated by an asterisk). Some habitat types are updated with high resolution layers (HRL, marked by a superscript +). Habitat types in bold where excluded from surrogacy analyses, because they represent highly artificial built-up areas (with weight = 0 in surrogacy analyses). (DOCX) [file pone.0251950.s007.docx]

**S3 Table** Habitat types included in prioritization analyses (sorted by ETE abbreviation). For each habitat type, the abbreviation, the habitat name, the geometric and thematic reliability values (1), range size and AUC of the Zonation performance curve are provided. Some habitat types were listed with several accuracy values, based on how often they were translated and used in the final EUNIS classification. We followed the ‘combined reliabilities’ approach (1) and took the maximum reliability value as a reference value (indicated by an asterisk). Some habitat types are updated with high resolution layers (HRL, marked by a superscript ^+^). Habitat types in bold where excluded from surrogacy analyses, because they represent highly artificial built-up areas (with weight=0 in surrogacy analyses).

| **habitat type** | **habitat name** | **geometric reliability** | **thematic reliability** | **own habitat code** | **range size (km^2^)** | **AUC** |
| --- | --- | --- | --- | --- | --- | --- |
| A100 | Littoral undetermined substrate with no sea ice presence | - | - | 41 | 68.36000 | 0.99772770 |
| A105 | Littoral sand with no sea ice presence | - | - | 42 | 0.30000 | 0.99983000 |
| A200 | Infralittoral undetermined substrate with no sea ice presence | - | - | 32 | 5.90000 | 0.99947803 |
| A205 | Infralittoral sand with no sea ice presence | - | - | 33 | 138.39000 | 0.99867043 |
| A206 | Infralittoral mud with no sea ice presence | - | - | 35 | 1.78000 | 0.99981548 |
| A306 | Circalittoral mud with no sea ice presence | - | - | 36 | 7.61000 | 0.99969668 |
| B1 | Coastal dunes and sandy shores | 5* | 7* | 34 | 5.10000 | 0.99955978 |
| B2 | Coastal shingle | 5* | 2* | 37 | 13.71000 | 0.99824092 |
| C1 | Surface standing waters | 9^+^ | 4^+^ | 6 | 1560.24000 | 0.91355051 |
| C2 | Surface running waters | 5 | 4 | 2 | 1597.55000 | 0.87395398 |
| C3 | Littoral zone of inland surface waterbodies | 6* | 5* | 19 | 1621.58000 | 0.94670255 |
| D1 | Raised and blanket bogs | 5* | 8* | 40 | 0.03000 | 0.99983000 |
| D4 | Base-rich fens and calcareous spring mires | 2* | 8* | 28 | 1.72000 | 0.99968006 |
| D5 | Sedge and reedbeds | 5* | 3* | 8 | 1249.69000 | 0.92751736 |
| D6 | Inland saline and brackish marshes and reedbeds | 5 | 5 | 38 | 0.68000 | 0.99979106 |
| E1 | Dry grasslands | 9^+^ | 2^+^ | 11 | 6374.13999 | 0.78522941 |
| E2 | Mesic grasslands | 6* | 4* | 4 | 29647.62997 | 0.50813865 |
| E3 | Seasonally wet and wet grasslands | 9^+^ | 3^+^ | 27 | 3118.05000 | 0.82308049 |
| E4 | Alpine and subalpine grasslands | 9^+^ | 8^+^ | 22 | 1586.63000 | 0.95684484 |
| E6 | Inland salt steppes | 9^+^ | 5^+^ | 30 | 115.01000 | 0.98268017 |
| E7 | Sparsely wooded grasslands | 9* | 7* | 16 | 25.43000 | 0.97878650 |
| F2 | Arctic | 7* | 8* | 23 | 452.03000 | 0.98696866 |
| F3 | Temperate and Mediterranean-montane scrub | 5* | 4* | 24 | 97.41000 | 0.99374123 |
| F4 | Temperate shrub heathland | 2 | 7 | 25 | 68.68000 | 0.99090658 |
| F5 | Maquis | 5* | 4* | 31 | 0.12000 | 0.99983000 |
| FB | Shrub plantations | 5* | 5* | 7 | 6060.00999 | 0.73127128 |
| G1 | Broadleaved deciduous woodland | 9^+^ | 4^+^ | 3 | 58187.91002 | 0.54242909 |
| G3 | Coniferous woodland | 9^+^ | 5^+^ | 14 | 13062.37999 | 0.81423318 |
| G4 | Mixed deciduous and coniferous woodland | 9^+^ | 5^+^ | 10 | 7558.81999 | 0.76408771 |
| G5 | Lines of trees | 5 | 5 | 5 | 2767.76999 | 0.81223679 |
| H2 | Screes | 5 | 2 | 13 | 347.36000 | 0.93751919 |
| H3 | Inland cliffs | 8* | 4* | 12 | 87.41000 | 0.99084328 |
| H5 | Miscellaneous inland habitats with very sparse or no vegetation | 5* | 3* | 17 | 110.99000 | 0.96018654 |
| I1 | Arable land and market gardens | 6* | 5* | 1 | 90784.89003 | 0.34219191 |
| I2 | Cultivated areas of gardens and parks | 5* | 5* | 26 | 130.16000 | 0.90673659 |
| **J1** | **Buildings of cities** | **9^+^** | **4^+^** | **9** | **-** | **-** |
| **J2** | **Low density buildings** | **9^+^** | **3^+^** | **15** | **-** | **-** |
| **J3** | **Extractive industrial sites** | **5** | **5** | **21** | **-** | **-** |
| **J4** | **Transport networks and other constructed hard-surfaced areas** | **5*** | **5*** | **18** | **-** | **-** |
| **J5** | **Highly artificial man-made waters and associated structures** | **9^+^** | **9^+^** | **20** | **-** | **-** |
| **J6** | **Waste deposits** | **5** | **5** | **29** | **-** | **-** |
| X2_3 | Coastal lagoons | 5 | 5 | 39 | 666.38000 | 0.98499521 |

*max. value of geometric and thematic reliability (among different classes)

^+^improved geometric and thematic reliability by using HRL

1. Weiss M, Banko G. Ecosystem Type Map v3. 1–Terrestrial and Marine Ecosystems. European Environment Agency (EEA)—European Topic Centre on Biological Diversity. 2018:79.
