# Supplementary material for: Limited reciprocal surrogacy of bird and habitat diversity and inconsistencies in their representation in Romanian protected areas
Source: PLoS One. 2022 Feb 11;17(2):e0251950. doi: 10.1371/journal.pone.0251950 (PMC8836316; doi:10.1371/journal.pone.0251950)
Supplement: S4 Table — For each species, breeding habitat, conservation status, range size and AUC of the Zonation performance curve are provided. (DOCX) [file pone.0251950.s008.docx]

**S4 Table** Bird species included in prioritization analyses, sorted by breeding habitat. For each species, breeding habitat, conservation status, range size and AUC of the Zonation performance curve are provided.

| **species** | **common name** | **breeding habitat** | **conservation status** | **range size** | **AUC** |
| --- | --- | --- | --- | --- | --- |
| *Accipiter brevipes* | Levant sparrowhawk | forest to (dense) woodland | least concern | 8061.57016 | 0.74990883 |
| *Accipiter gentilis* | Northern goshawk | forest to (dense) woodland | least concern | 77534.98407 | 0.59119630 |
| *Accipiter nisus* | Eurasian sparrowhawk | forest to (dense) woodland | least concern | 90978.51613 | 0.58160768 |
| *Acrocephalus agricola* | Paddyfield warbler | wetlands and shores | least concern | 121.65638 | 0.99784597 |
| *Acrocephalus arundinaceus* | Great reed warbler | wetlands and shores | least concern | 39035.58315 | 0.59233947 |
| *Acrocephalus palustris* | Marsh warbler | arable land, open woodland to grassland | least concern | 43637.63396 | 0.54630492 |
| *Acrocephalus schoenobaenus* | Sedge warbler | wetlands and shores | least concern | 12035.75636 | 0.73894781 |
| *Acrocephalus scirpaceus* | Eurasian reed warbler | wetlands and shores | least concern | 6736.38939 | 0.86876331 |
| *Aegithalos caudatus* | Long-tailed tit | forest to (dense) woodland | least concern | 57872.27831 | 0.59468770 |
| *Alauda arvensis* | Eurasian skylark | arable land, open woodland to grassland | least concern | 87559.61642 | 0.48238245 |
| *Alcedo atthis* | Common kingfisher | wetlands and shores | least concern | 9597.15719 | 0.81748182 |
| *Anas platyrhynchos* | Mallard | wetlands and shores | least concern | 63027.82016 | 0.57953751 |
| *Anas strepera* | Gadwall | wetlands and shores | least concern | 2874.35555 | 0.92502952 |
| *Anser anser* | Greylag goose | wetlands and shores | least concern | 2058.04150 | 0.92071810 |
| *Anthus campestris* | Tawny pipit | arable land, open woodland to grassland | least concern | 50424.37797 | 0.50528700 |
| *Anthus spinoletta* | Water pipit | arable land, open woodland to grassland | least concern | 968.85526 | 0.87441015 |
| *Anthus trivialis* | Tree pipit | arable land, open woodland to grassland | least concern | 63217.79347 | 0.58182476 |
| *Aquila pomarina* | Lesser spotted eagle | arable land, open woodland to grassland | least concern | 55005.77685 | 0.58658109 |
| *Asio otus* | Long-eared owl | arable land, open woodland to grassland | least concern | 61755.49970 | 0.55281792 |
| *Athene noctua* | Little owl | generalist and close to humans | least concern | 51542.02505 | 0.56806267 |
| *Bonasa bonasia* | Hazel grouse | forest to (dense) woodland | least concern | 10857.52089 | 0.69164888 |
| *Bubo bubo* | Eurasian eagle-owl | arable land, open woodland to grassland | least concern | 14583.92646 | 0.69569954 |
| *Burhinus oedicnemus* | Eurasian stone-curlew | arable land, open woodland to grassland | least concern | 8085.43081 | 0.71756006 |
| *Buteo buteo* | Common buzzard | arable land, open woodland to grassland | least concern | 116307.14361 | 0.54823333 |
| *Buteo rufinus* | Long-legged buzzard | arable land, open woodland to grassland | least concern | 34836.83075 | 0.55854096 |
| *Calandrella brachydactyla* | Greater short-toed lark | arable land, open woodland to grassland | least concern | 7761.67829 | 0.47625058 |
| *Caprimulgus europaeus* | European nightjar | arable land, open woodland to grassland | least concern | 40532.75741 | 0.61605196 |
| *Carduelis cannabina* | Common linnet | arable land, open woodland to grassland | least concern | 52845.10863 | 0.59140200 |
| *Carduelis carduelis* | European goldfinch | generalist and close to humans | least concern | 86726.94063 | 0.58135138 |
| *Carduelis chloris* | European greenfinch | generalist and close to humans | least concern | 70295.94246 | 0.60629425 |
| *Carduelis spinus* | Eurasian siskin | forest to (dense) woodland | least concern | 2839.96055 | 0.85193304 |
| *Certhia brachydactyla* | Short-toed treecreeper | forest to (dense) woodland | least concern | 2013.28922 | 0.83382209 |
| *Certhia familiaris* | Eurasian treecreeper | forest to (dense) woodland | least concern | 44435.41463 | 0.60044900 |
| *Charadrius alexandrinus* | Kentish plover | wetlands and shores | least concern | 402.80379 | 0.98700321 |
| *Charadrius dubius* | Little ringed plover | wetlands and shores | least concern | 11437.19756 | 0.76026444 |
| *Cinclus cinclus* | White-throated dipper | wetlands and shores | least concern | 9042.78134 | 0.65070329 |
| *Coccothraustes coccothraustes* | Hawfinch | forest to (dense) woodland | least concern | 50998.87105 | 0.57640894 |
| *Columba livia domestica* | Domestic pigeon | generalist and close to humans | least concern | 54317.06182 | 0.56837325 |
| *Columba oenas* | Stock dove | arable land, open woodland to grassland | least concern | 21185.92176 | 0.57768825 |
| *Coracias garrulus* | European roller | arable land, open woodland to grassland | least concern | 24555.93468 | 0.58215295 |
| *Coturnix coturnix* | Common quail | arable land, open woodland to grassland | least concern | 83034.01921 | 0.50421482 |
| *Crex crex* | Corn crake | arable land, open woodland to grassland | least concern | 50193.95260 | 0.54882257 |
| *Cuculus canorus* | Common cuckoo | arable land, open woodland to grassland | least concern | 120210.34997 | 0.54176096 |
| *Cygnus olor* | Mute swan | wetlands and shores | least concern | 7537.50276 | 0.87129993 |
| *Delichon urbicum* | Common house martin | generalist and close to humans | least concern | 43626.40116 | 0.62783735 |
| *Dendrocopos leucotos* | White-backed woodpecker | forest to (dense) woodland | least concern | 22250.06326 | 0.58679011 |
| *Dendrocopos major* | Great spotted woodpecker | forest to (dense) woodland | least concern | 78391.42973 | 0.59522897 |
| *Dendrocopos medius* | Middle spotted woodpecker | forest to (dense) woodland | least concern | 32955.40140 | 0.61298939 |
| *Dendrocopos minor* | Lesser spotted woodpecker | forest to (dense) woodland | least concern | 38934.26162 | 0.63192121 |
| *Dendrocopos syriacus* | Syrian woodpecker | generalist and close to humans | least concern | 50665.64839 | 0.60917411 |
| *Dryocopus martius* | Black woodpecker | forest to (dense) woodland | least concern | 54417.02676 | 0.61511955 |
| *Emberiza cia* | Rock bunting | arable land, open woodland to grassland | least concern | 2271.28839 | 0.76424958 |
| *Emberiza cirlus* | Cirl bunting | arable land, open woodland to grassland | least concern | 1344.30056 | 0.83244702 |
| *Emberiza citrinella* | Yellowhammer | arable land, open woodland to grassland | least concern | 73230.19592 | 0.56465259 |
| *Emberiza hortulana* | Ortolan bunting | arable land, open woodland to grassland | least concern | 46165.78880 | 0.48603792 |
| *Emberiza melanocephala* | Black-headed bunting | arable land, open woodland to grassland | least concern | 16282.96982 | 0.42695403 |
| *Emberiza schoeniclus* | Common reed bunting | wetlands and shores | least concern | 5717.80150 | 0.87258025 |
| *Erithacus rubecula* | European robin | forest to (dense) woodland | least concern | 73091.24488 | 0.59193831 |
| *Falco subbuteo* | Eurasian hobby | arable land, open woodland to grassland | least concern | 80250.96856 | 0.57867657 |
| *Falco tinnunculus* | Common kestrel | arable land, open woodland to grassland | least concern | 101869.92984 | 0.52123089 |
| *Ficedula parva* | Red-breasted flycatcher | forest to (dense) woodland | least concern | 9690.38547 | 0.59766073 |
| *Ficedula semitorquata* | Semicollared flycatcher | forest to (dense) woodland | least concern | 187.86025 | 0.98597321 |
| *Fringilla coelebs* | Common chaffinch | forest to (dense) woodland | least concern | 84296.54300 | 0.58662739 |
| *Fulica atra* | Eurasian coot | wetlands and shores | least concern | 10633.35631 | 0.84990775 |
| *Galerida cristata* | Crested lark | arable land, open woodland to grassland | least concern | 40207.25027 | 0.56719285 |
| *Gallinula chloropus* | Common moorhen | wetlands and shores | least concern | 14865.47427 | 0.78560910 |
| *Garrulus glandarius* | Eurasian jay | forest to (dense) woodland | least concern | 78083.09732 | 0.58596724 |
| *Hieraaetus pennatus* | Booted eagle | arable land, open woodland to grassland | least concern | 21325.78723 | 0.65608778 |
| *Himantopus himantopus* | Black-winged stilt | wetlands and shores | least concern | 7601.28856 | 0.82358760 |
| *Hippolais icterina* | Icterine warbler | forest to (dense) woodland | least concern | 4734.55775 | 0.76345323 |
| *Hippolais pallida* | Eastern olivaceous warbler | arable land, open woodland to grassland | least concern | 636.41807 | 0.88715480 |
| *Hirundo rustica* | Barn swallow | generalist and close to humans | least concern | 92684.57095 | 0.54812916 |
| *Ixobrychus minutus* | Little bittern | wetlands and shores | least concern | 9672.45646 | 0.83022420 |
| *Lanius collurio* | Red-backed shrike | arable land, open woodland to grassland | least concern | 105283.62519 | 0.53238743 |
| *Lanius minor* | Lesser grey shrike | arable land, open woodland to grassland | least concern | 49545.80656 | 0.56142450 |
| *Locustella fluviatilis* | River warbler | wetlands and shores | least concern | 9883.75889 | 0.68186192 |
| *Locustella luscinioides* | Savi's warbler | wetlands and shores | least concern | 13024.20543 | 0.78439736 |
| *Loxia curvirostra* | Red crossbill | forest to (dense) woodland | least concern | 12968.37490 | 0.73620076 |
| *Lullula arborea* | Woodlark | arable land, open woodland to grassland | least concern | 53795.41696 | 0.59298697 |
| *Luscinia luscinia* | Thrush nightingale | arable land, open woodland to grassland | least concern | 13674.82363 | 0.57008231 |
| *Luscinia megarhynchos* | Common nightingale | arable land, open woodland to grassland | least concern | 51973.65397 | 0.58210337 |
| *Melanocorypha calandra* | Calandra lark | arable land, open woodland to grassland | least concern | 10370.97087 | 0.46776981 |
| *Merops apiaster* | European bee-eater | arable land, open woodland to grassland | least concern | 56258.58013 | 0.60406792 |
| *Motacilla alba* | White wagtail | generalist and close to humans | least concern | 80438.03822 | 0.61047234 |
| *Motacilla cinerea* | Grey wagtail | wetlands and shores | least concern | 23852.95797 | 0.61261186 |
| *Motacilla flava* | Western yellow wagtail | arable land, open woodland to grassland | least concern | 59462.76328 | 0.46563968 |
| *Nucifraga caryocatactes* | Spotted nutcracker | forest to (dense) woodland | least concern | 7249.30683 | 0.77635845 |
| *Oenanthe isabellina* | Isabelline wheatear | arable land, open woodland to grassland | least concern | 1217.57581 | 0.88971704 |
| *Oenanthe oenanthe* | Northern wheatear | arable land, open woodland to grassland | least concern | 45487.63231 | 0.59425191 |
| *Oriolus oriolus* | Eurasian golden oriole | forest to (dense) woodland | least concern | 89862.92670 | 0.56965290 |
| *Otus scops* | Eurasian scops owl | arable land, open woodland to grassland | least concern | 55827.45031 | 0.57201477 |
| *Parus caeruleus* | Eurasian blue tit | forest to (dense) woodland | least concern | 62848.64603 | 0.59909183 |
| *Parus cristatus* | European crested tit | forest to (dense) woodland | least concern | 8947.17371 | 0.74419417 |
| *Parus lugubris* | Sombre tit | arable land, open woodland to grassland | least concern | 3738.45119 | 0.74668054 |
| *Parus major* | Great tit | generalist and close to humans | least concern | 104681.26292 | 0.56798917 |
| *Parus montanus* | Willow tit | forest to (dense) woodland | least concern | 14259.97792 | 0.69191956 |
| *Parus palustris* | Marsh tit | forest to (dense) woodland | least concern | 51336.50316 | 0.56590091 |
| *Passer domesticus* | House sparrow | generalist and close to humans | least concern | 62745.54466 | 0.57630376 |
| *Passer hispaniolensis* | Spanish sparrow | arable land, open woodland to grassland | least concern | 14484.03502 | 0.58587226 |
| *Phoenicurus ochruros* | Black redstart | generalist and close to humans | least concern | 41834.92825 | 0.61907826 |
| *Phylloscopus collybita* | Common chiffchaff | forest to (dense) woodland | least concern | 71431.24855 | 0.59370802 |
| *Phylloscopus sibilatrix* | Wood warbler | forest to (dense) woodland | least concern | 25237.17963 | 0.59518699 |
| *Pica pica* | Eurasian magpie | arable land, open woodland to grassland | least concern | 99061.75709 | 0.51677079 |
| *Picoides tridactylus* | Eurasian three-toed woodpecker | forest to (dense) woodland | least concern | 6997.02729 | 0.76442532 |
| *Picus canus* | Grey-headed woodpecker | forest to (dense) woodland | least concern | 54437.01940 | 0.60940213 |
| *Picus viridis* | European green woodpecker | arable land, open woodland to grassland | least concern | 52897.80818 | 0.59607385 |
| *Podiceps cristatus* | Great crested grebe | wetlands and shores | least concern | 7371.90259 | 0.85279854 |
| *Prunella collaris* | Alpine accentor | arable land, open woodland to grassland | least concern | 235.86942 | 0.99085294 |
| *Prunella modularis* | Dunnock | arable land, open woodland to grassland | least concern | 5153.02586 | 0.77200520 |
| *Pyrrhula pyrrhula* | Eurasian bullfinch | forest to (dense) woodland | least concern | 7230.06574 | 0.76103579 |
| *Rallus aquaticus* | Water rail | wetlands and shores | least concern | 5556.81137 | 0.86233389 |
| *Recurvirostra avosetta* | Pied avocet | wetlands and shores | least concern | 3202.83744 | 0.86916668 |
| *Regulus regulus* | Goldcrest | forest to (dense) woodland | least concern | 15427.99648 | 0.69644053 |
| *Saxicola rubetra* | Whinchat | arable land, open woodland to grassland | least concern | 29109.11443 | 0.52158472 |
| *Saxicola torquatus* | African stonechat | arable land, open woodland to grassland | least concern | 52718.00252 | 0.55167082 |
| *Serinus serinus* | European serin | generalist and close to humans | least concern | 9720.81702 | 0.66615576 |
| *Sitta europaea* | Eurasian nuthatch | forest to (dense) woodland | least concern | 64966.35194 | 0.57578479 |
| *Streptopelia decaocto* | Eurasian collared dove | generalist and close to humans | least concern | 55746.31333 | 0.56628207 |
| *Streptopelia turtur* | European turtle dove | arable land, open woodland to grassland | least concern | 62021.97272 | 0.59983583 |
| *Strix aluco* | Tawny owl | forest to (dense) woodland | least concern | 53225.06837 | 0.57489893 |
| *Strix uralensis* | Ural owl | forest to (dense) woodland | least concern | 32850.95119 | 0.59425224 |
| *Sturnus vulgaris* | Common starling | generalist and close to humans | least concern | 100143.48935 | 0.54204224 |
| *Sylvia atricapilla* | Eurasian blackcap | forest to (dense) woodland | least concern | 83074.98241 | 0.59172058 |
| *Sylvia communis* | Common whitethroat | arable land, open woodland to grassland | least concern | 89801.92144 | 0.50865592 |
| *Sylvia curruca* | Lesser whitethroat | arable land, open woodland to grassland | least concern | 83056.52531 | 0.59982587 |
| *Sylvia nisoria* | Barred warbler | arable land, open woodland to grassland | least concern | 28279.00220 | 0.66816209 |
| *Tachybaptus ruficollis* | Little grebe | wetlands and shores | least concern | 15069.00180 | 0.69765523 |
| *Tadorna ferruginea* | Ruddy shelduck | wetlands and shores | least concern | 1481.95172 | 0.90693381 |
| *Tadorna tadorna* | Common shelduck | wetlands and shores | least concern | 1028.39293 | 0.93484062 |
| *Troglodytes troglodytes* | Eurasian wren | forest to (dense) woodland | least concern | 38787.66051 | 0.55674206 |
| *Turdus merula* | Common blackbird | generalist and close to humans | least concern | 76184.82882 | 0.58633076 |
| *Turdus philomelos* | Song thrush | forest to (dense) woodland | least concern | 71071.01128 | 0.59438408 |
| *Turdus torquatus* | Ring ouzel | arable land, open woodland to grassland | least concern | 2106.73183 | 0.85863618 |
| *Turdus viscivorus* | Mistle thrush | arable land, open woodland to grassland | least concern | 48748.68883 | 0.56797913 |
| *Tyto alba* | Western barn owl | arable land, open woodland to grassland | least concern | 13425.57259 | 0.55469077 |
| *Upupa epops* | Eurasian hoopoe | arable land, open woodland to grassland | least concern | 64782.17531 | 0.60267177 |
| *Vanellus vanellus* | Northern lapwing | arable land, open woodland to grassland | least concern | 44779.79424 | 0.53426956 |
