# Supplementary material for: Limited reciprocal surrogacy of bird and habitat diversity and inconsistencies in their representation in Romanian protected areas
Source: PLoS One. 2022 Feb 11;17(2):e0251950. doi: 10.1371/journal.pone.0251950 (PMC8836316; doi:10.1371/journal.pone.0251950)
Supplement: S5 Table — (DOCX) [file pone.0251950.s009.docx]

**S5 Table** AUC values for all the performance curves within each of the surrogacy analyses.

| Bird species as surrogate | | Habitat types as surrogate | |
| --- | --- | --- | --- |
| Curve | AUC | Curve | AUC |
| Surrogate: Bird species | 0.6563234 | Surrogate: Habitat types | 0.901305 |
| Target: Habitat types | 0.7418273 | Target: Bird species | 0.5609637 |
| Optimal habitat curve | 0.901305 | Optimal bird curve | 0.6563234 |
| Random habitat curve | 0.4992973 | Random bird curve | 0.4846996 |
