# Supplementary material for: Limited reciprocal surrogacy of bird and habitat diversity and inconsistencies in their representation in Romanian protected areas
Source: PLoS One. 2022 Feb 11;17(2):e0251950. doi: 10.1371/journal.pone.0251950 (PMC8836316; doi:10.1371/journal.pone.0251950)
Supplement: S1 File — (DOCX) [file pone.0251950.s010.docx]

**S1 Supporting information**

**Bird species distribution modeling**

To model species distributions for the forthcoming Romanian Breeding Bird Atlas ((1), in preparation), all presence data available for Romania from the years 2006-2017 (occasional observations included) recorded in the breeding season were used. Presence records were obtained from Ornitodata (2), OpenBirdMaps (3) and Rombird (4). These databases and corresponding tools have been designed and are managed by the Romanian Ornithological Society / BirdLife Romania and Milvus Group, the two partner organizations developing the atlas and modeled distribution maps. The databases contain all existing bird data of the two organizations, collected during systematic monitoring schemes, atlas work, and different censuses, but also comprise occasional observations. Although many species occurrences were thus collected systematically, all data were treated as occasional observations in the modeling process. To reduce sampling bias caused by unequal coverage and the use of occasional observations, occurrence data were resampled. From every 1x1 km grid cell only one observation was used, and the remaining data were further rarefied by selecting a maximum of five presences in every grid cell of the ETRS89 LAEA 10x10 km grid. The final median number of occurrences used in modeling was 818 (S1 Table). To further reduce the effects of sampling bias, a bias file was created by computing the kernel density of all observations, and using the inverse density as weights for the occurrence data.

To describe habitat conditions in Romania, an initial set of 172 environmental variables was used, which included climate, vegetation, soil, and topographic characteristics, as well as land cover types (S2 Table). Climate variables were obtained from the CliMond repositories (5), percent habitat cover data from Corine Land Cover 2012 (6) and the Ecosystem types of Europe from EUNIS (European Nature Information System; (7)), and the priority habitats ‘forest’, ‘sub-alpine’ and ‘alpine’ in Romania (8). Information on forest management was acquired from the forest management map of European forests (9), pedological data were extracted from the European Soil Atlas (10), and remote sensing data on vegetation from the Copernicus Global Land Service (11). Furthermore, variables describing open areas, shrubs, woodlands and wetlands in surrounding areas, and distance to water at the landscape level were derived from Corine Land Cover 2012 (6).

To reduce redundancy and autocorrelation between the environmental variables, pairs of variables with a Pearson correlation coefficient > 0.8 were identified, and the one with the highest correlations with other variables was excluded. The final data set consisted of 73 variables (S2 Table).

Species distributions were modeled using MaxEnt 3.4.1 (12) at 1 km resolution, with the exception of twelve species with large home ranges or where the number of records was relatively low (S1 Table). For these species, models were created at 2 km resolution. For each species,

100 models were initially run with default parameters and subsequently averaged.

Model accuracy was assessed using the area under the Receiver Operator Curve (AUC), where values range between 0 and 1, with 0.5 representing a model that is no better than random, and values > 0.7 generally considered to indicate good model performance. The data was split randomly in 70% training and 30% test data to further evaluate performance. A visual evaluation of the models was performed based on expert opinion. This model assessment process was paired with the selection of one of the threshold values offered by selection of one of the threshold values offered by MaxEnt (14) to truncate the resulting maps. Species were considered to be absent below this threshold, with corresponding values set to 0. Because the thresholds are case specific and hard to select based only on calculation only (15), threshold selection was done for each species using expert opinion by the Romanian Atlas Committee, considering the omission errors and the species’ distribution. For ten species, the number of occurrences used in modeling was close to that of the environmental variables, and overfitting was a potential issue. For species where model evaluation based on expert opinion suggested overfitting – interestingly those with few records were not among them - , model complexity was reduced by increasing the regularization multiplier from 1 to 2 (S1 Table) (13). New models with this setting were not considered to be overfitting.

**Reliability values of habitat types**

The habitat types used in this study are a result of a complex mapping approach by the European Environment Agency (EEA) (7) and combine different data sets, such as the non-spatially referenced EUNIS (European Nature Information System) habitat classification scheme and the spatially explicit and Corine-based “Mapping and Assessment of Ecosystem and their services (MAES)” ecosystem classes scheme (16).

In order to assess the accuracy of the habitat mapping, the EEA provides reliability values and maps (see Tables 5.2, 5.4 and Maps 6.3 in (16)). These reliability values are divided into geometric (a measure of the spatial resolution) and thematic (a measure of the accuracy of the thematic classification) reliability. The reliability highly depends on the input data set (resolution and information content) and ranges from one to ten, where one constitutes a very low reliability and ten a very high reliability (Table 5.2 in (16)). A value of five constitutes an intermediate geometric or thematic reliability and is often observed. We included the reliability values in table S3 based on the documented values in the technical note of this data set. For some habitat classes, several reliability values were present, depending on how often this particular class is occurring in higher hierarchy classes. For example, the habitat class E2 which describes ‘mesic grassland’ is occurring in three different higher classes such a ‘Pastures’, ‘Complex cultivation patterns’ and ‘Land principally occupied by agriculture with significant areas of natural vegetation’. In these cases, we report the maximum reliability values as a measure of accuracy. Some of the habitat classes underwent an improvement of accuracy by incorporating high resolution layers (HRL) and were highlighted accordingly in table S3. For the first six habitat classes (Table S3) no reliability values were reported, yet they comprise only a very small fraction of the total land surface area in Romania.

**References**

1. Fântânâ C, Kovács I. The Romanian breeding bird atlas 2006-2017, a common scheme of Milvus Group Association and the Romanian Ornithological Society. in preparation. 2020.

2. Ornitodata. Ornitodata 2018 [Available from: <https://ornitodata2.sor.ro/ornitodata>.

3. MilvusGroup. OpenBirdMaps: An online database for bird distribution and abundance in Romania. 2018.

4. Rombird. Rombird 2018 [Available from: <https://www.rombird.ro>.

5. Kriticos DJ, Webber BL, Leriche A, Ota N, Macadam I, Bathols J, et al. CliMond: global high‐resolution historical and future scenario climate surfaces for bioclimatic modelling. Methods Ecol Evol. 2012;3(1):53-64.

6. European Environment Agency. CORINE Land Cover (CLC) 2012 2017 [Available from: <https://land.copernicus.eu/pan-european/corine-land-cover>.

7. European Environment Agency. Mapping and Assessing the Condition of Europe's Ecosystems: Progress and Challenges. 2019.

8. Stăncioiu P, Lazăr G, Tudoran G, Candrea Bogza Ş, Predoiu G, Şofletea N. Habitate prioritare alpine, subalpine şi forestiere din România–Masuri de gospodărire. Editura Universităţii Transilvania din Brasov. 2008.

9. Hengeveld GM, Nabuurs G-J, Didion M, van den Wyngaert I, Clerkx A, Schelhaas M-J. A forest management map of European forests. Ecology and Society. 2012;17(4).

10. Van Ranst E. Soil Atlas of Europe. European Soil Bureau Network, European Commission, Office for Offical Publications of the European Communities; 2005.

11. Buchhorn M, Smets B, Bertels L, De Roo B, Lesiv M, Tsendbazar N-E, et al. Copernicus Global Land Service: Land Cover 100m: Version 3 Globe 2015-2019: Product User Manual. 2020.

12. Phillips S, Dudík M, Schapire R. Maxent software for modeling species niches and distributions v. 3.4. 1. URL: <http://biodiversityinformatics> amnh org/open_source/maxent. 2017.

13. Radosavljevic A, Anderson RP. Making better Maxent models of species distributions: complexity, overfitting and evaluation. Journal of biogeography. 2014;41(4):629-43.

14. Philips SJ, Dudík M, Schapire RE. Maxent software for modeling species niches and distributions (Version 3.4.1) 2017 [Available from: <http://biodiversityinformatics.amnh.org/open_source/maxent/>.

15. Liu C, White M, Newell G. Selecting thresholds for the prediction of species occurrence with presence‐only data. Journal of biogeography. 2013;40(4):778-89.

16. Weiss M, Banko G. Ecosystem Type Map v3. 1–Terrestrial and Marine Ecosystems. European Environment Agency (EEA)—European Topic Centre on Biological Diversity. 2018:79.
